# Strategies for Early Keratoconus Diagnosis: A Narrative Review of Evaluating Affordable and Effective Detection Techniques

**DOI:** 10.3390/jcm14020460

**Published:** 2025-01-13

**Authors:** Arige Gideon Abou Said, Joan Gispets, Einat Shneor

**Affiliations:** 1 Department of Optometry and Vision Science, Hadassah Academic College, Jerusalem 9101001, Israel; arigeab@edu.hac.ac.il; 2 Department of Optics and Optometry, Universitat Politècnica de Catalunya, Violinista Vellsolà, 37, 08222 Terrassa, Spain; joan.gispets@upc.edu

**Keywords:** keratoconus, early keratoconus, diagnosis

## Abstract

Keratoconus is a progressive corneal disorder that can lead to irreversible visual impairment if not detected early. Despite its high prevalence, early diagnosis is often delayed, especially in low-to-middle-income countries due to limited awareness and restricted access to advanced diagnostic tools such as corneal topography, tomography, optical coherence tomography, and corneal biomechanical assessments. These technologies are essential for identifying early-stage keratoconus, yet their high cost limits accessibility in resource-limited settings. While cost and portability are important for accessibility, the sensitivity and specificity of diagnostic tools must be considered as primary metrics to ensure accurate and effective detection of early keratoconus. This review examines both traditional and advanced diagnostic techniques, including the use of machine learning and artificial intelligence, to enhance early diagnosis. Artificial intelligence-based approaches show significant potential for transforming keratoconus diagnosis by improving the accuracy and sensitivity of early diagnosis, especially when combined with imaging devices. Notable innovations include tools such as SmartKC, a smartphone-based machine-learning application, mobile corneal topography through the null-screen test, and the Smartphone-based Keratograph, providing affordable and portable solutions. Additionally, contrast sensitivity testing demonstrates potential for keratoconus detection, although a precise platform for routine clinical use has yet to be established. The review emphasizes the need for increased awareness among clinicians, particularly in underserved regions, and advocates for the development of accessible, low-cost diagnostic tools. Further research is needed to validate the effectiveness of these emerging technologies in detecting early keratoconus.

## 1. Introduction

Keratoconus is a progressive corneal disease that affects both eyes asymmetrically [1,2,3,4,5], leading to impaired vision. Early diagnosis of keratoconus is crucial since collagen cross-linking can prevent the disease from progressing [6,7,8] and potentially avoid the need for more invasive treatments such as corneal transplantation [1,9]. Despite the high prevalence of keratoconus in many countries [10], many patients remain undiagnosed [11]. Therefore, affordable detection techniques should be deployed in screening studies and in primary eye care. Advanced stages of the disease are relatively easy to identify [8]; the challenge is to diagnose eyes with keratoconus at early stages [12].

Many people with keratoconus are unaware that they have the disease, resulting in delayed treatment and an increased risk of irreversible visual impairment. For example, in two different population-based studies, in Israel [13,14], 40% and 60% of participants were unaware of their keratoconus status despite having recently seen an ophthalmologist or optometrist. Another study in Iran showed [15] that 42% of keratoconus subjects were unaware of their condition. Therefore, the best way to identify patients with early keratoconus may be via population-based screening, particularly in regions of the world where the prevalence is high.

In the Middle East, the prevalence has been shown to be over 2.0% [10,13,14,16,17,18,19,20]. Similarly, in India, the prevalence was found to be 2.3%, and in China, it was found to be 1.1% [21,22]. It should be noted that all these studies are population-based. The lower rates found in some studies [23,24,25,26] may stem from the fact that they were conducted in hospitals or clinics, and thus suffer from an ascertainment bias: people with mild keratoconus may not present to secondary and tertiary centers [27]. Furthermore, people with known risk factors for keratoconus should be closely monitored for the development of the disease. These include being a first-degree relative of a subject with keratoconus, [17,26], or being the offspring of families with consanguinity [13,14,18,19,20,21,28,29].

While clinical signs enable the clinician to detect intermediate and advanced keratoconus easily, the detection of early keratoconus and forme fruste remains a challenge [30].

This review aims to evaluate various methods for the early detection of keratoconus, providing a comprehensive overview of the current diagnostic approaches. The article includes traditional techniques, as well as advanced methods that incorporate machine learning/artificial intelligence, offering insights into the latest advancements in the field and their potential to improve early diagnosis.

## 2. Materials and Methods

### 2.1. Method of Literature Review

We conducted a literature review using two databases, PubMed and Google Scholar, covering publications available up to October 2024. The search strategy employed combinations of the following keywords: “detecting”, “early keratoconus”, “subclinical keratoconus”, “form fruste keratoconus”, “retinoscopy”, “keratometer”, “ophthalmoscope”, “slit lamp bio-microscopy”, “corneal hysteresis”, “corneal biomechanics”, “artificial intelligence”, and “machine learning”. Studies that answered the review objectives were included. We also reviewed the reference lists of relevant articles to identify additional studies that may have been missed in our initial search. Our review addresses different methods for the detection of keratoconus at early stages, with a focus on the suitability for screening and identification of the condition. Studies were excluded if they were not peer-reviewed or not published in English, and papers that focused on general keratoconus diagnosis without specific emphasis on early-stage detection were excluded.

### 2.2. Definitions

This review will assess early keratoconus (KC) detection based on affordability, required expertise, patient cooperation, and diagnostic performance in terms of sensitivity/specificity.

The early stages of keratoconus are often referred to as subclinical, forme fruste keratoconus, or keratoconus suspect, though there is a lack of unified criteria in the use of these terms [31]. These stages are typically characterized by a topographic pattern consistent with keratoconus but without apparent clinical signs [5,17,32].

For the purpose of this review, studies using any of these terms will be included, and all will be collectively referred to as “early keratoconus”.

The affordability of instruments was categorized into three levels [11,33]: unaffordable instruments, which are typically available only in tertiary clinics; moderately affordable instruments, which are usually found in secondary care and primary care settings; and affordable methods, which are commonly used in primary care clinics.

The expertise required to perform the technique is divided into three categories: clinician (optometrist or ophthalmologist), trained technician, and layperson.

Instruments that use validated indices to automatically generate diagnoses are considered to be low-skill instruments. Some instruments have the potential for telemedicine, while others require clinical skills for interpretation of test results.

The level of patient cooperation required was evaluated, with some techniques demanding high patient cooperation (i.e., focus on a specific point with a wide-open eye), while others require minimal cooperation and are faster. Sensitivity and specificity and patient cooperation are based on scientific literature when possible.

The terms “sensitivity” and “specificity” are commonly used to describe the performance characteristics of diagnostic tests or screening tools. Sensitivity quantifies the ability of a screening test to correctly identify individuals who have the target condition or disease [34]. The specificity of a test is defined as the ability of a test to correctly identify individuals who do not have the target condition or disease (true negatives) [34].

A technique that is defined as suitable for community screening should be simple, portable, and affordable to enable access to early-stage screening [35].

In this review, we examined studies that included both artificial intelligence and machine learning, treating the two terms interchangeably due to the overlap in their usage and the difficulty in clearly distinguishing between them in the literature.

## 3. Results

**Figure 1:**
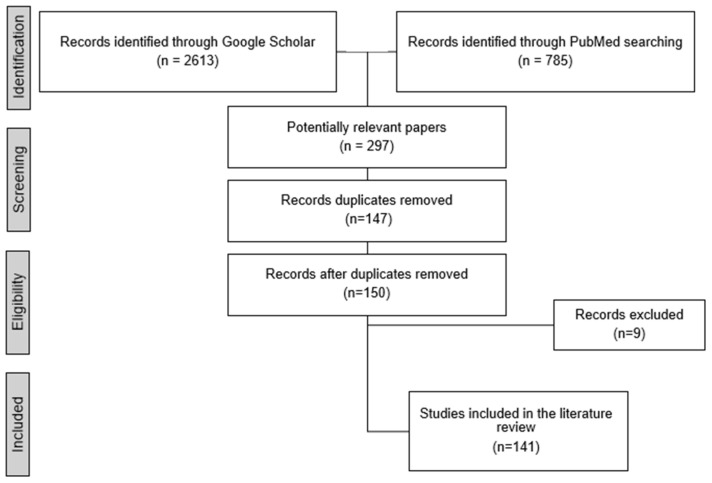
Filtering steps for study inclusion in this review.

The literature search yielded a total of 2613 articles and papers from Google Scholar and 758 from PubMed. Following an initial screening of titles and abstracts, 297 potentially relevant articles were selected for further review. Duplicate studies identified across PubMed and Google Scholar (*n* = 147) were removed. Additionally, articles that were not peer-reviewed (*n* = 6) and those not published in English (*n* = 3) were excluded. This process resulted in the inclusion of 141 articles that met the eligibility criteria and were incorporated into the review (Figure 1). These articles were analyzed to provide a comprehensive overview of methods for detecting early-stage keratoconus, with a particular focus on their suitability for screening and identification.

### 3.1. Instruments in Secondary and Tertiary Clinics

There are several instruments used in the diagnosis of keratoconus that are typically found only in tertiary centers due to affordability issues. These instruments include corneal topography and tomography, optical coherence tomography (OCT), and corneal biomechanical parameter testing.

The Placido-based corneal topography is a non-invasive imaging technique that measures the anterior surface of the cornea, providing quantitative data on curvature and surface irregularities through computational algorithms [36,37,38,39]. While corneal topographers do not require specialized clinical skills [40], technicians need training for accurate image acquisition. Although most devices offer indices to identify abnormal corneas and suggest keratoconus diagnoses, studies indicate that results are more reliable when performed by experienced clinicians [41,42]. Interpreting results may still require clinical expertise [40], as corneal irregularities can lead to misleading topographic maps, complicating the differentiation of keratoconus from conditions such as contact lens-induced warpage [43], poor tear film quality, or lid artifacts [44]. Placido-based topography mainly analyzes the central anterior corneal surface [12], potentially missing posterior elevation abnormalities characteristic of early keratoconus [32]. Therefore, the technique alone may not be sufficient to detect the early stages of the disease [12,45]. While videokeratography indices often detect early keratoconus, they are inadequate to diagnose cases that are not yet clinically apparent [45,46,47], with a modest classification rate for potential keratoconus [46] and widely varying sensitivity and specificity across parameters [38,48,49,50,51,52] (11–100%/5–100%, respectively, Table 1). Although corneal topographers have been widely used as a community screening tool, they do have several limitations. Image acquisition in non-optimal conditions (such as dry eye syndrome, narrow palpebral apertures, long eyelashes, or nystagmus) can affect the accuracy of measurements and diagnoses [53], especially in early cases of keratoconus [32]. Poor repeatability, particularly in young children (where poor cooperation and poor ability to focus exist) can also negatively impact the quality of the image captured [54]. Additionally, the high cost and limited portability of corneal topographers hinder their large-scale deployment [35]. Furthermore, in many countries, these instruments are only found in tertiary centers and are not available for large-scale screening.

**Table 1:** Instrument utility assessment for detecting early keratoconus.

Technique	Cost	Need for Clinical Skills to Perform	Need of Clinical Interpretation Skills	Patient Cooperation (Interaction)	Study	Sensitivity and Specificity	Community Screening
Corneal tomography	High	Trained technician [40]	Medium [55,56]	High [35,57]	[41,45,49,50,52,58,59,60,61,62,63,64,65,66]	Range: 57–98%/29–100% Specific values: Sib: 67.7–82%/85–100% [50,52,67,68] light backscatter: 90%/95% [61] KVb: 74%/72% [52] ThkMin: 92%/45% [52] CCT: 91%/46% [52] BAD-D: 70–98%/32–85% [60,61,63,64,67,68,69] BCVb: 64.5%/97.7% [68] BAD-Dt: 87%/29% [60] BAD-Da: 74.3–80%/35% [60] BAD-PImax: 93%/47% [60] SDP: 57–89%/81–86% [49] PRFI: 71.7–97.4%/84.7–87.9% [47,67] Combination of morpho-geometric, volumetric and clinical parameters: 96.8%/94.5% [41] PPI min + CH: 80%/80% [64] ARTmax: 83.3%/74.3% [70] CKI: 0.27–77.3%/41.3–97.7% [65] IHD: 75–83.3%/60.3–88.6% [65,68] PE: 53–95.5%/66.7–95.4% [65,68] RMS, and RMS/A KVb and the apex front curvature: Showed high sensitivity for differentiating early KC [71] Combined parameters (I-S, SteepK-OppK, PostKmax-Position Y, Pr/Ar, PTI2): 99%/99% [72] RMS HOA: 70%/69.77% [69] B-Ele-Thin: 70%/70.54% [69] PPI avg: 73.33–77.4%/70.4–73.64% [68,69] Da: 70–73.6%/70.54–88.3% [69,73] TPpach. 87.0%/71.4% [70]	No
Corneal topography	High	Trained technician [40]	Low [40]	High [53]	[38,45,46,48,49,50,51,52,58]	Range: 11–100%/5–100% Specific values: AAI: 77–94%/67–97% [49] CSI: 22–97%/5–100% [50] DSI: 54.1%/69.8% [48] IHA 67–83.3%/0.5–86.3% [65,68] I-S: 11–81%/79–91% [48,49,51] ISV: 74.5–100%/61.8–96% [48,50,65] IVA: 10.8%/95% [48] KISA%: 60%/100% [38] Kavg: 63–85%/52–74% [45,52] KI: 86.4–100%/63.5–100% [48,65,68] KPI: 57%/58–84% [49] OSI: 22–84%/45–99% [49,50] Sif: 74–95%/76% [45,50,52,67] SAI: 43–44%/91–92% [48,49] SRI: 68–83%/51–86% [48,49,67] Rmin: 69.8%/61.4% [65]	No
OCT	High	Trained technician [74]	High [12]	High [75]	[58,66,76,77,78,79,80]	Range: 48–90%/88–94% Specific values: Fourier Posterior indices asymmetry: 58%/88% [76] Fourier Posterior indices Higher-order: 48%/94% [76] Several OCT parameters sensitivity: 90% [78] The coincident thinning (CTN) index 93% [81] As/Ps: 92%/96% [47]	No
Corneal biomechanical parameters and Hysteresis measurements	High	Trained Technician *	High *	High *	[58,64,66,82,83,84,85,86]	Biomechanics improve diagnosis [82,84] ΔDAR2: 88.9%/NA [87] ΔIR: 88.4%/NA [87] ΔMax ICR: 80.5%/NA [87] ΔSP-A1: 76.2%/NA [87] SP-A1: 82.1%/74.4% [68] SP-A1: 84.93%/33.33% [88] TBI: 70.8–99%/67–95.4% [47,88,89] A1 dArc Length: 86.6%/84.4% [73] HC-Radius: 83.5%/80.5% [73] A2 Time: 83.5%/76.3% [73] CBI: 67.7–78.08%/71–97.7% [68,73,88] SSIv1: 88.14%/27.14% [90] SSIv2: 79.38%/93.47% [90] DA ratio: 73.97%/47.83% [88] ARTh: 78.08%/79.71% [88]	No
Slit lamp biomicroscopy	medium	Clinician [35]	High [91]	High [91]	[92,93]	Not tested, but A positive association between the presence of clinical signs and topographic parameters [92] No clear clinical signs in slit lamp examination [93]	No ^£^
Keratometry	medium	Clinician [94,95]	High [96]	High [97]	[39,66,94,98]	Miss inferior steepening [66] limitations in providing information about the corneal topography beyond the points of measurements [98]	No
Retinoscopy	Low	Clinician [95,99]	High [99]	Low [99]	[66,95,99]	98%/80% Scissoring reflex is sensitive for detecting early stages of KC 98%/80% [95,99]	Yes
Ophthalmoscopy	Low	Clinician [8]	High but has potential for telemedicine *	Low [8]	[8]	Not tested Sensitive in detecting early KC with the ability to classify the stage of the disease. On going research	Yes
Smart-phone based technologies	Low	Layperson [35]	High but has potential for telemedicine *	Low [35]	[35,100,101,102,103]	Not tested SmartKC [100]; SBK [35]; the null-screen test method; On going research	Yes
Contrast sensitivity	Low	Trained technician	High [104]	High *	[105,106,107,108,109]	Not tested CS can help in detecting and grading keratoconus in different severity and even with good visual acuity	yes

^£^ (unless portable device used). * Not evidence based. Colors: Colors used in the table indicate levels based on cost, clinical interpretation skills, and patient cooperation. Color code: Green: Low cost, minimal need for clinical interpretation skills, and low patient cooperation. Yellow: Medium cost, moderate need for clinical interpretation skills, and moderate patient cooperation. Red: High cost, significant need for clinical interpretation skills, and high patient cooperation. Regarding the need for clinical skills to perform the test: Green: Test can be performed by a layperson. Yellow: Trained technician required to perform the test. Red: Clinician needed to perform the test. Notes: Abbreviations: KC, keratoconus; AAI, asphericity asymmetry index; SAI, surface asymmetry index; SRI, surface regularity index; CSI, center/surround index; DSI, differential sector index; IHA, index of height asymmetry; I-S, inferior–superior index; ISV, index of surface variance; IVA, index of vertical asymmetry; KISA%, keratoconus percentage index; Kavg, average keratometry value; KI, keratoconus index; KPI, keratoconus probability index; OSI, opposite sector index; Sif, symmetry index front; Sib, symmetry index back; SAI, surface asymmetry index; SRI, surface regularity index; KVb, keratoconus vertex back; ThkMin, minimum corneal thickness; CCT, central corneal thickness; BAD-D, Belin–Ambrosio-enhanced ectasia display total deviation value; BAD-Dt Belin–Ambrosio-enhanced ectasia display thinnest point; BAD-Da Belin–Ambrosio-enhanced ectasia display thinnest point displacement; BAD-PImax, Belin–Ambrosio-enhanced ectasia display; pachymetric progression index maximum; SDP, standard deviation of corneal power index; PPI Min minimum pachymetric progression index; CH, corneal hysteresis; OCT, optical coherence tomograph; SBK, smartphone-based keratography; CS, contrast sensitivity. RMS, root mean square: RMS/A, root mean square/area; (KVf, KVb) elevation at KC vertex; SteepK-OppK, steepest K–opposed K; PostKmax, posterior steepest keratometry; Pr/Ar, ratio between the steepest posterior radius and the steepest anterior radius; PTI, percentage of thickness increase; ΔDAR2, change in dynamic apex radius 2; ΔIR, change in inward radius; ΔMax ICR, change in maximum intra-corneal resistance; ΔSP-A1, change in stiffness parameter A1; TBI, tomographic and biomechanical index, HC-radius, radius of curvature of the corneal apex at the highest concavity; A2, time taken to reach the second applanation; CBI, corvis biomechanical index; Da, deformation amplitude; PRFI, Pentacam random forest index; PE, posterior elevation(microns); KI, keratocononus index; IHA, index of height asymmetry; IHD, index of height decentration; BCVb, baiocchi-calossi-versaci back; SP-A1, stiffness parameter at first applanation; ARTh, ambrósio relational thickness to the horizontal profile; SSIv2, stress–strain index (version 2); TPpach, thinnest point pachymetry; CKI, central keratoconus index; KI, keratoconus index; Rmin, minimum radius of curvature; B-Ele-Thin, back elevation at the thinnest location; ARTmax, ambrósio relational Thickness; RMS HOA, root mean square of higher-order aberration; Da, deviation of normality of ambrỏsio relational thickness; DA Ratio, the ratio between DA measured at the apex and 2 mm from the center of the cornea.

According to the consensus established by Gomes et al. in 2015 [32], corneal tomography is widely considered the gold standard for identifying early keratoconus. This non-invasive imaging technique is capable of accurately mapping both the anterior and posterior surfaces of the cornea, providing high-precision results. Its sensitivity allows detection of early signs of keratoconus, making it the most reliable method for early diagnosis [5,32,41,45,46,47,49,50,52,58,60,61,62,63,65,66,110,111]. Furthermore, the indices obtained through corneal tomography enable accurate severity staging of the disease, and its results are easily interpretable. Performing corneal tomography requires a trained technician [40]. However, an experienced examiner with clinical skills will yield a more reliable assessment when interpreting the results [55,56]. Different indices showed different ranges of sensitivity and specificity (between 57–90%/29–100%, respectively [49,50,52,60,61,63,64,71], Table 1). The Belin–Ambrosio-enhanced ectasia display (BAD), which is available on Pentacam, has a strong diagnostic performance [5,32,41,45,46,47,49,50,52,58,60,61,62,63,65,66,110,111] and is widely used to differentiate normal corneas from abnormal ones and even eyes with the potential of developing the disease [69,78,112]. However, a combination of several tomography indices, along with a comprehensive evaluation of the patient’s clinical picture [65] and other clinical parameters, increases sensitivity and specificity [41,64]. Due to their high cost, corneal tomography instruments are typically only available at tertiary centers, with few clinics equipped with such technology. Furthermore, corneal tomography instruments are not portable, need clinical skills to interpret the results [55,56], and require patient cooperation, which limits their use in large-scale community screening [35,57]. These limitations pose significant challenges to the widespread adoption of corneal tomography as a tool for large-scale screening programs and are not a viable option in many countries or regions [35].

Optical coherence tomography (OCT) is a high-resolution imaging technique that uses near-infrared light and low-coherence interferometry to provide detailed information on tissue morphology, including thickness maps of the individual corneal layers [76,113]. This imaging modality can generate both anterior and posterior topography, as well as cross-sectional images of the cornea [114]. Various types of OCT have been developed and proved to be effective in detecting early KC [5,32,41,45,46,47,49,50,52,58,60,61,62,63,65,66,110,111], allowing for the identification of small corneal shape irregularities [72] and thickness variations, as well as subtle changes that may indicate early disease onset [115,116,117]. One example is polarization-sensitive optical coherence tomography (PS-OCT), which measures how light splits into two beams when passing through the organized tissue of the cornea. This non-invasive method can detect early structural changes in the cornea [45,46,47], aiding in the early diagnosis of keratoconus [12,77,78,79,80,81]. Moreover, research has unveiled the presence of a distinctive “epithelial doughnut pattern” observable through OCT imaging in individuals afflicted with keratoconus. This phenomenon is attributed to an epithelial compensatory mechanism wherein the epithelial layer appears to undergo structural adjustments aimed at rectifying irregularities within the corneal layers [118,119,120]. While epithelial mapping can be useful for diagnosing keratoconus in general, its effectiveness is limited, particularly for detecting eyes with the potential of having the disease since it could diagnose 40% of the cases when used alone [121]. Therefore, corneal epithelial thickness mapping using OCT is useful for early diagnosis [122] when combined with other imaging techniques [47,115,116,121] and/or artificial intelligence methods [121,123].

OCT is an expensive and generally non-portable diagnostic device that requires technician training for proficient generation of corneal images [12]. Furthermore, its dependency on patient cooperation [75] and its limited effectiveness when used alone for detecting early keratoconus highlight the need to complement it with other imaging techniques or AI-based methods to enhance diagnostic accuracy [47,115,116,121].

Corneal biomechanical parameters and corneal hysteresis: Corneal biomechanical properties refer to the measurable characteristics that describe how the cornea responds to external forces [124]. These properties are crucial for the early detection of keratoconus [51]. Two commercial instruments frequently used in clinical practice to assess corneal biomechanics are the corneal visualization Scheimpflug technology (Corvis ST) and the ocular response analyzer (ORA) [125,126,127].

The Corvis ST, a non-contact air-puff tonometer, combines Pentacam data for integrated analysis of corneal shape and biomechanics. It captures high-speed images of the eye to monitor corneal deformation [51]. Research shows that the Corvis ST is highly effective in evaluating corneal biomechanics, particularly in distinguishing keratoconus, early keratoconus, and normal eyes. However, its accuracy in detecting early keratoconus is slightly lower compared to the Sirius corneal tomographer and the Pentacam [51], and its clinical application is still limited [67]. A recent study suggests two key modifications to improve the Corvis ST device’s ability to detect keratoconus: rotating the device’s camera by 90 degrees during clinical examinations, particularly in the nasal-inferior quadrant where keratoconus often develops, and incorporating asymmetry-based analyses by evaluating additional sectional images. These adjustments could enhance the device’s effectiveness in detecting keratoconus, even in cases where topographic analysis does not reveal obvious signs [128].

The ORA instrument measures corneal biomechanics, including corneal hysteresis (CH) and corneal resistance factor (CRF). While these parameters have shown limited sensitivity and specificity for diagnosing early keratoconus [66], the overall efficacy of corneal hysteresis measurement in this context remains uncertain. Some studies have found that corneal hysteresis can differentiate between early keratoconus and healthy controls [84,86,87,89,129,130], even in the absence of topographical abnormalities [131,132,133]. Conversely, some studies have indicated that corneal hysteresis has low sensitivity and specificity unless used in conjunction with other indices or models [64,82,84,134] (Table 1). However, when tomography, biomechanics, and artificial intelligence methods are combined, they can significantly improve the precision and reliability of early detection [47,66,73,83,135]. Nonetheless, identifying early KC remains a challenge and requires further technological advancements [51,136].

The measurement of corneal hysteresis requires a trained technician, as well as an understanding of corneal biomechanics to interpret the results. The high cost and requirement for expertise and patient cooperation have limited the widespread utilization of these instruments.

Motion-tracking Brillouin microscopy is an emerging noninvasive 3D mapping technique that measures the Brillouin frequency shift to estimate the cornea’s viscoelastic properties without corneal deformation. It shows promise for detecting biomechanical changes in keratoconus and evaluating corneal crosslinking efficacy [137]. However, its clinical use faces challenges, including sensitivity to eye movements and the need for faster data acquisition [126]. Further technological advancements are needed to enhance its practicality and accuracy for early keratoconus diagnosis.

Unfortunately, the unaffordability of these techniques presents a significant barrier to the diagnosis and monitoring of keratoconus, particularly in low-to-low-middle income-countries where access to contemporary diagnostic and therapeutic equipment is limited [35].

### 3.2. Primary Eye-Care Clinics

This section will address the diagnosis of early keratoconus with tools often found in primary eye-care clinics. These tools are generally affordable but require clinical skill in performance and in interpretation of the results.

The keratometer is a tool that requires the user to align mires to the patient’s eye to measure the minimum and maximum keratometry (K) readings and their corresponding axes. This is achieved by focusing on four points within the 3.0–4.0 mm central corneal zone [138]. The process of focusing and alignment requires clinical skill to perform and patient cooperation [94,95,96,97]. The keratometer is a cost-effective tool, which may be suitable for community-based screening. Furthermore, manual keratometry provides a qualitative visualization of mires, which is useful in diagnosing manifest keratoconus [94]. The disadvantage is that it may not be a good tool for the identification of early keratoconus [38,39,66,139] in patients who do not have steep anterior corneas. In addition, since measurements are limited to the central anterior curvature of the cornea, it may not detect manifest keratoconus with cones offset from the center of the cornea [94] or pellucid marginal degeneration. Since these patients are good candidates for cross-linking, this is a considerable limitation. According to Hashemi et al. [98], manual keratometry exhibits comparable repeatability to other advanced imaging modalities for manifest keratoconus, but its reliability depends on the user’s experience, which should have high clinical expertise [94].

Slit lamp biomicroscopy is a comprehensive exam that involves using a microscope with a narrow beam of light to examine the cornea. It is often used to diagnose advanced keratoconus as it can reveal any structural abnormalities found with advanced disease in the cornea, such as Munson sign [140], Vogt striae, and Fleischer rings [5,141,142,143]. Advantages include its ability to provide detailed images of the cornea and its accessibility in most primary eye-care clinics. Disadvantages include the need for trained eye-care professionals to perform and interpret the results of the exam and the need for patient cooperation [91]. Furthermore, in early stages of the condition, structural abnormalities may not be present or may be very subtle [92,93].

### 3.3. Primary Eye-Care Diagnosis with Affordable and Portable Equipment

Several low-cost tools for detecting keratoconus are currently in development and testing, using different methods such as ophthalmoscopy [8] and contrast sensitivity testing [144] to detect early signs of keratoconus. While these methods offer affordability and portability, their effectiveness relies heavily on examiner skills, and they may not match the sensitivity and specificity of advanced technologies like corneal topography. However, if successfully implemented, these tools have the potential to enable extensive screening initiatives and are widely used in low- and middle-income countries and could prove to be a valuable asset for eye care practitioners without access to advanced technology.

The retinoscope is a non-invasive, affordable, handheld instrument that is used to measure the refractive error of the eye. In keratoconus, the cornea becomes irregularly shaped, causing distorted light reflection, and a scissoring reflex is observed [4,5,95,143]. Several studies have shown that the retinoscope proves to have high sensitivity and moderate specificity in detecting keratoconus (when compared to Pentacam) [95,99], especially in cases of mild, intermediate, and advanced stages [92,99,140,145]. The retinoscope can be particularly useful for testing children or non-compliant patients, such as individuals with Down’s syndrome [99], because it does not require patient cooperation. However, it has limitations in accurately diagnosing and classifying the disease in its early stages [92,99]. Another disadvantage is that using the retinoscope is challenging and necessitates clinical skills to perform and interpret the results [95,99].

Ophthalmoscopy is a non-invasive, inexpensive, widely available, and handheld tool that examines the retina without the need for high patient cooperation. Some studies have shown that ophthalmoscopy can detect signs of keratoconus [141,146,147,148,149] but may not be as sensitive and/or specific as other tests. In addition, the use of ophthalmoscopy requires clinical skills. Recently, Gideon Abou Said et al. [8] revisited the oil droplet sign in keratoconus and its utility for early diagnosis and screening using an ophthalmoscope attached to an iPhone. The study found that the oil droplet sign (annular dark shadow, ADS) was a good tool for detecting early keratoconus [8]. This sign was present in all 37 eyes with keratoconus and 13 eyes of early disease, but in none of the 37 control eyes. The study also found a correlation between the height of the sign and the severity of keratoconus. This tool is superior to retinoscopy in terms of its ability to classify the stages of the disease but, like retinoscope, it requires skill to perform [8]. Nonetheless, the output can be photographed and analyzed automatically for easy interpretation, so either a trained technician or even a layperson can perform the test.

Due to the affordability and portability of the technique, it could be an excellent tool for community-based screening. However, the sensitivity and specificity of this tool have not been tested on a large population.

SmartKC is a low-cost, smartphone-based tool that uses machine-learning algorithms to detect keratoconus [100,101]. The system comprises a 3D-printed cone-shaped Placido ring attachment on a smartphone’s camera and an off-the-shelf USB blue-colored LED light strip to project concentric black-and-blue rings on the human eye. The captured corneal images are analyzed by SmartKC’s software, which uses an image processing pipeline to generate assessments that are similar to clinical gold-standard topographers. The SmartKC system was evaluated in a clinical study involving 101 eyes, achieving a high sensitivity of 94.1% and a specificity of 100% [100]. SmartKC is relatively low cost, portable, does not require specialized clinical skill or patient cooperation, and it has the potential to be widely available to patients who may not have access to specialized eye care. However, it is still in the early stages of development and has not yet been widely tested.

The null-screen test method [102,103] uses a small conical null screen for corneal topography attached to the camera of a mobile device for performing topography measurements. By comparing the actual image of the corneal surface with the ideal image produced by the null screen, it is possible to determine any deviations from a perfect surface and, thus, obtain accurate measurements of corneal topography. The null screen is an artificial intelligence-based tool that is an affordable and cost-effective tool, and it does not require specialized clinical skill or patient cooperation to measure the shape of aspherics and free-form surfaces. It is a non-contact test and does not require specially designed optics, making it a cheap and easy technique to implement. Specifically, the null screen remains in the early stages of development and has yet to be subjected to widespread testing. Consequently, it is unclear whether the null screen can effectively replace corneal tomography or detect early-stage keratoconus. Furthermore, the sensitivity and specificity of the null screen have not been evaluated, which represents a crucial limitation in terms of its clinical application. Therefore, further investigation is warranted to determine the feasibility and accuracy of the null screen as a diagnostic tool for keratoconus.

Similarly, the smartphone-based keratograph (SBK; EMAGine AG) [35] is a cost-effective and portable Placido-based topography tool that employs artificial intelligence and smartphone processing power to detect keratoconus. While it has not been validated, it presents an attractive solution since it is affordable, portable, and specifically designed to be user-friendly for unskilled workers, enabling them to easily administer it outside of a clinical setting with the needs of minimal patient cooperation [35].

In addition, contrast sensitivity has been found to be significantly lower for keratoconus subjects when compared with healthy controls [106,107,108]. This was also true for keratoconus patients who presented good “best corrected visual acuity” [105]. Therefore, contrast sensitivity may be a useful tool for screening or even detecting and monitoring keratoconus. However, no simple and precise platform currently exists for detecting keratoconus based on contrast sensitivity, and further studies are needed to develop and validate portable, affordable devices for this purpose.

Although low-cost tools are essential for improving accessibility in underserved regions, it is crucial to validate their diagnostic performance in terms of sensitivity and specificity. Tools with inadequate diagnostic accuracy may lead to false positives or missed cases, limiting their clinical utility for early keratoconus detection.

### 3.4. Integration of Artificial Intelligence Methods for Improving Early Detection Keratoconus

Several low-cost tools for detecting keratoconus are currently in development, and artificial intelligence (AI) methods have gained traction in medical fields, including ophthalmology, for improving diagnostic accuracy across various pathologies [150,151,152,153,154,155]. While modern ophthalmic instruments, such as tomographers and topographers, include built-in software with advanced computational algorithms, it is essential to distinguish these pre-trained, task-specific algorithms from the broader application of AI in clinical practice. In this context, “AI” refers to advanced algorithms capable of analyzing and integrating multiple diagnostic parameters—such as corneal topography, tomography, and biomechanical assessments—to improve accuracy and reduce variability in detecting early keratoconus. Unlike embedded algorithms in devices like SmartKC or Keratograph, which are pre-configured for specific applications, modern AI methods employ iterative training on large datasets to continually refine and optimize diagnostic capabilities. In the following section, we present the effectiveness of AI methods in early keratoconus detection, including their integration with advanced imaging technologies and a summary of recent findings.

In the context of early keratoconus detection, AI methods have demonstrated enhanced accuracy, with high sensitivity and specificity, particularly when integrated with imaging technologies such as corneal topography, tomography, and biomechanical assessments [47,68,123,156,157,158,159]. A systematic meta-analysis reported a pooled sensitivity of 0.88 for early keratoconus detection using machine-learning methods [160], with some neural networks achieving sensitivities and specificities exceeding 90%. However, the study did not differentiate between early and established cases, limiting insights into early detection accuracy [161]. Table 2 provides a comprehensive summary of findings from the meta-analysis supplemented by additional recent studies, highlighting the sensitivity and specificity of various diagnostic tools for early keratoconus detection. The table includes studies from the past 5 years, focusing on various methods for detecting early keratoconus. These methods include artificial intelligence (AI) approaches such as machine learning and deep learning, as well as traditional image processing and analysis techniques, selected through a systematic search. These studies were identified through a systematic search incorporating the following keywords, “artificial intelligence” and “machine learning”, added to the original search strategy. Of the 141 articles included in the review, 25 met these specific inclusion criteria. Studies that were already covered in the meta-analyses or reviews were excluded from the table. However, individual studies published in the last 5 years, which addressed early keratoconus detection and were not part of any review, were included. Only studies that provided data on sensitivity and specificity for early keratoconus detection were considered for inclusion. As a result, a total of 20 studies met these criteria and were included in Table 2.

The table provides an overview of sensitivity and specificity ranges for various AI-based tools in keratoconus detection. Sensitivity across studies varied widely, from as low as 28.5% [85,161] to as high as 100% [123,151,157,158,162], with specificity ranging from 14% [85] to 100% [85,123,151,157,158,162,163]. Many studies reported high sensitivity and specificity levels, with several reaching above 90%, indicating strong diagnostic potential. Some studies also reported high accuracy or AUC (area under the curve) values [67,123,135,150,153,154,156,157,158,159,162,163,164,165,166,167,168], spanning from 0.575 to nearly 1.0, reinforcing AI’s capability for reliable keratoconus detection.

However, variability in sensitivity and specificity across models and datasets suggests that while AI offers promising diagnostic accuracy, consistency may vary based on specific model types, imaging devices, and data quality. For instance, certain models achieved nearly perfect specificity and sensitivity, while others were more modest, indicating room for optimization in identifying early or potential keratoconus cases. This variability highlights the potential for AI to significantly impact early diagnosis but also underscores the need for standardization and validation to ensure reliability across different clinical settings.

**Table 2:** Summary of studies on machine-learning methods for early keratoconus detection.

Author, Year	Study Type	Number of Papers That Are Specified for Ekc (Years of Data Included in the Study)	Name of Included Devices/Imaging Modality	Type of Data	Sensitivity%	Specificity%	AUC/Accuracy%
Hashemi et al., 2024 [156]	A systemic and meta-analysis review	22 (Up to March 2022)	Pentacam Sirius Galilei Tomy Orbscan CORVIS OCT OPD-Scan	Tomographic Topographic Aberrometric Biomechanics	70.8–100	84.95–99.7	0.92–0.999/85.3–99.7
Afifah et al., 2024 [161]	A systematic review and meta-analysis	6 (2018–2023)	Pentacam Pentacam-HR TMS-4 UHR-OCT	Tomographic Topographic	97 ^£^	96–98 ^£^	NA
Bodmer et al., 2024 [163]	A systematic review and exploratory meta-analysis	7 (Up to February 2022)	Pentacam TMS-4	Tomographic Topographic	93.7–95.1	94.4–100	0.99/86.4–98.9
Goodman and Zhu, 2024 [154]	A systemic review	24 (Up to October 2023)	Pentacam HR Pentacam Sirius Orbscan OPD-Scan III SD-OCT UHR -OCT OCT (CASIA) Corvis ST Air-puff Slit lamp	Tomographic Topographic Aberrometric Biomechanics Clinical findings Demographic VA Geometric Tonometric	75–98	89.8–97.9	0.81–0.99/68.7–99.78
Nguyen et al., 2024 [158]	A narrative review	13 (1997–2024)	Pentacam-HR Galilei Sirius TMS-1 MS-39 Corvis-ST AS-OCT SD-OCT MS-39	Tomographic Topographic Biomechanics Tonometric	41.3–100	40.5–100	0.57–0.98/93–100
Hashemian et al., 2024 [167]	Comprehensive review	4 (*)	Pentacam ORA Corvis ST	Tomographic Topographic Biomechanics	80–85.2%	90–96.6%	0.945
Tey et al., 2024 [166]	Review	17 (Up to August 2023)	Pentacam Galilei Sirius Oculyzer TMS-1 TMS-4 SmartKC Corvis ST APT SD-OC AS-OCT OPD-Scan III	Tomographic Topographic Aberometric Biomechanics Tonometric	71.5–100	83.97–100	0.80–0.99/ 88.7–100
Huo et al., 2024 [150]	Review	11 (June 2013 to September 2022)	ORA Corvis ST Pentacam AS-OCT SD-OCT	Tomographic Topographic Biomechanics Abberometric Tonometric	75–100	82.07–100	NA/83.33–99.6
Niazi et al., 2023 [67]	A systematic narrative review	18 (Up to October 2022)	Pentacam Pentacam HR Orbscan II Sirius Corvis ST Air puff SD-OCT	Tomographic Topographic Biomechanics Genetic data Tonometric	66.6–100	70–100	0.81–0.99/89–98.7
Vandevenne et al., 2023 [151]	A systematic review	28 (2013–2022)	Pentacam Sirius Orbscan IIz Orbscan Galilei TMS-1 ARK-1 Corvis-ST OCT (CASIA, RCTVue)	Tomographic Topographic Biomechanics	47–100	54–100	NA
Zhang et al., 2023 [162]	A systematic review	18 (1997–2022)	Pentacam Sirius Galilei Orbscan IIz TMS-1 TMS-4 MS-39 OPD Scan III Corvis-ST AS-OCT (CASIA) UHR-OCT	Tomographic Topographic Abberometric Biomechanics	76.92–100	83.1–100	0.96–1.0/85.4–100
Cao et al., 2022 [160]	A systematic review and meta-analysis	17 (1995–2020)	Pentacam HR Orbscan IIz Sirius Galilei TMS-4 ORA Corvis ST UHR-OCT OCT (CASIA)	Tomographic Topographic Biomechanics	82.2–92.3	91.4–96.7	NA
Shanthi et al., 2022 [159]	A systematic review	14 (2010–2020)	Pentacam Sirius Galilei Corvis ST OPD Scan III VX120 OCT (CASIA)	Tomographic Topographic Abberometric Biomechanics Geometric Demographic	63–97.59	82–98.72	0.69–0.98/88.8–98.2
Kang et al., 2022 [165]	Systematic review	7 (2020–2022)	Tomography * Topography * AS-OCT	Tomographic Topographic	99–86	99–85	0.995–0.93/69–99
Maile et al., 2021 [85]	A systematic review	26 (2012–2020)	Pentacam Pentacam HR Orbscan IIz Sirius Galilei Corvis-ST TMS-4 MS-39 OCT (RCTVue, SS-1000 CASIA) OPD scan	Tomographic Topographic Abberometric Biomechanics Demographic	28.5–98.5	14–100	NA
Jiang et al., 2024 [164]	Multicenter diagnostic study		Pentacam HR	Tomographic Topographic	98	98	0.96/98
Yang et al., 2024 [157]	Retrospective case-control study		Corvis-ST	Biomechanics	100	75–100	0.92–1.00/ 85–95
Mourgues et al., 2024 [123]	Retrospective case-control study		SS-OCT (CASIA 2)	Tomographic Topographic Pachymetric Abberometric	100 84	100 90	0.98–0.99/NA
Ren et al., 2023 [168]	Case-control study		Pentacam Corvis ST	Tomographic Topographic Biomechanics	76.9	90.4	0.91/NA
Chen et al., 2023 [153]	Prospective diagnostic study		Corvis-ST	Tomographic Topographic Biomechanics UDVA, CDVA Demographic Refraction Tonometric Slit lamp Fundus Examination	70.30–75.25	89.4–99.7	0.88–0.89/ 86.3–93.4

* Not specified. ^£^ Specified only for two studies. Notes: Abbreviations: HR, high resolution; OPD, optical path difference; OCT, optical coherence tomography; AS, anterior segment; SD, spectral domain; UHR, ultra-high resolution; SS, swept source; RCTVue, retina comprehensive total vue; TMS, tomey corneal topographer system; SmartKC, smart keratoconus detection system; ORA, ocular response analyzer; APT, air-puff tonometry; ARK, automated keratometer; UDVA, uncorrected distance visual acuity; CDVA, corrected distance visual acuity; VA, visual acuity, NA, not available.

## 4. Discussion

Currently, as summarized in Table 1, an optimal modality for community-based screening of early keratoconus does not exist. Instruments such as corneal tomographers and OCTs are highly specific and sensitive for the detection of early keratoconus but expensive and not portable. More accessible tools, such as keratometry and retinoscopy, are affordable and portable yet lack sufficient sensitivity for early keratoconus detection. Although several novel methods hold the potential to be both affordable and sensitive, they have not been extensively tested for community-based screening applications.

Portable tools like the retinoscope and ophthalmoscope could offer practical solutions for early keratoconus screening, particularly in remote or underserved areas, thus improving public health accessibility. However, these tools may not be sensitive enough to detect early cases. Furthermore, they have not yet been widely utilized in population-based studies for this purpose.

Recently, new tools have been developed to address this challenge. Gideon Abou Said et al. [8] demonstrated that the annular dark shadow observed with the ophthalmoscope can effectively detect early keratoconus and classify its stages [8]. This technique shows promise as a practical, low-cost alternative for keratoconus staging, complementing traditional methods like retinoscopy. Integrating automated image processing or machine learning with the annular dark shadow technique could further enhance its sensitivity and specificity. By eliminating the need for a trained clinician, this approach may enable automated screening for early keratoconus in community settings. This further highlights the potential of the annular dark shadow technique as a viable option for early detection in resource-limited settings, where advanced diagnostic tools may not be available. Future studies focusing on real-world validation analysis will be crucial to demonstrate its clinical utility.

In addition, the healthcare field has increasingly adopted artificial intelligence (AI) algorithms for automated early keratoconus screening, which achieve high sensitivity and specificity with minimal human intervention. These AI-driven systems integrate multiple clinical parameters from diverse instruments, enhancing diagnostic accuracy [66,85,169]. The performance of these artificial intelligence methods tools is comparable to that of experienced corneal specialists and can ease the workload on healthcare professionals, making the technology accessible to non-specialists [85,170]. Continued development and validation of these AI methods could significantly impact keratoconus screening and early treatment options, bridging the gap in community-level screening tools and enhancing early detection outcomes.

This review has several limitations. Emerging technologies in the early stages may be absent from peer-reviewed journals, leading to their exclusion. The narrative review approach introduces subjectivity, as study selection and interpretation may vary. Limiting the search to PubMed and Google Scholar, peer-reviewed papers, and English-language journals may have excluded relevant studies, further restricting the generalizability of findings across diverse healthcare settings and populations. Furthermore, Table 2 is limited by the lack of homogeneous inputs across studies, making direct comparisons of methodologies and outcomes challenging. Future research should utilize a broader range of databases for greater comprehensiveness.

## 5. Conclusions

In summary, the early diagnosis of keratoconus is crucial for effective management, preventing the progression of the disease and preserving vision. Studies indicate that keratoconus often progresses significantly during childhood and puberty, potentially leading to severe visual impairment. However, diagnosing the condition in this age group is challenging due to factors such as affordability and variability in test reliability, which can be affected by limited cooperation and difficulty maintaining focus during testing procedures. Future research should focus on enhancing the sensitivity and specificity of portable, low-cost tools to ensure their reliability for large-scale screening programs. With the advancements in diagnostic tools and techniques, including artificial intelligence, eye care professionals will be able to detect and treat keratoconus at an earlier stage and younger age, resulting in a better quality of life. Developing a screening tool for detecting early keratoconus can save vision and avoid expensive and penetrating procedures. While expensive tools may be of use in high-income countries, since they do not require a trained optometrist or ophthalmologist to perform or interpret, there is still a need for portable and affordable equipment that can be used in large-scale screening projects. Furthermore, low-cost portable instruments may offer significant benefits in remote areas, where unspecialized examiners could perform tests and interpret clinical results. Several low-cost tools for detecting keratoconus are currently in development and testing. If successfully implemented, these tools have the potential to improve awareness of the disease by initiating extensive screening in low- and middle-income regions and could prove to be a valuable asset for clinicians without access to advanced technology. However, the challenge lies in convincing the industry to prioritize the adaptation of existing technology rather than investing in even more sophisticated solutions. Moreover, as clinicians, we have a social responsibility to advocate for solutions that are accessible to everyone, regardless of the economic situation of their countries. This includes influencing companies to develop sensory devices that prioritize affordability, portability, and simplicity as key factors for success. As proposed by Hafezi and Hafezi [35], companies should consider shifting their business models towards a sales volume-based approach rather than solely targeting a limited number of elite users. By promoting these values, we can contribute to making these devices feasible and accessible for a larger global market.

## Data Availability

No new data were created or analyzed in this study. Data sharing is not applicable to this article.
